# The Association between Advanced Liver Fibrosis and Mortality Is Modified by Dietary Quality among Korean Adults: Results from the Korea National Health and Nutrition Examination Survey with Mortality Data

**DOI:** 10.3390/nu15061501

**Published:** 2023-03-21

**Authors:** Juhee Lee, Garam Jo, Dahyun Park, Hee Ju Jun, Jae Hyun Bae, Min-Jeong Shin

**Affiliations:** 1 Interdisciplinary Program in Precision Public Health, Graduate School, Korea University, Seoul 02841, Republic of Korea; 2 Department of Internal Medicine, Korea University Anam Hospital, Korea University College of Medicine, Seoul 02841, Republic of Korea; 3 School of Biosystems and Biomedical Sciences, College of Health Science, Korea University, Seoul 02841, Republic of Korea

**Keywords:** diet, healthy, fibrosis, liver cirrhosis, nonalcoholic fatty liver disease, mortality

## Abstract

Advanced fibrosis in nonalcoholic fatty liver disease (NAFLD) is associated with an increased risk of mortality; however, an independent association of liver fibrosis with mortality is not well defined. We aimed to investigate the association between advanced liver fibrosis and all-cause and cardiovascular mortality and the mediation effect of diet quality. We analyzed 35,531 participants with suspected NAFLD, excluding competing etiologies of chronic liver disease, from the Korea National Health and Nutrition Examination Survey 2007–2015, and followed up until 31 December 2019. The severity of liver fibrosis was assessed using the NAFLD fibrosis score (NFS) and the fibrosis-4 index (FIB-4). The Cox proportional hazards model was used to examine the association of advanced liver fibrosis with mortality. During a mean 8.1 years of follow-up, 3426 deaths occurred. Advanced liver fibrosis determined by NFS and FIB-4 was associated with increased risks of all-cause and cardiovascular mortality after adjusting for confounders. When NFS and FIB-4 were combined, the high NFS + high FIB-4 group was significantly associated with higher risks of all-cause mortality (hazard ratio [HR] 1.85, 95% CI 1.42–2.43) and cardiovascular mortality (HR 2.04, 95% CI 1.23–3.39), respectively, compared with the low NFS + low FIB-4 group. However, these associations were attenuated in people with high diet quality. Advanced liver fibrosis is an independent risk factor for all-cause and cardiovascular mortality in people with NAFLD, and the association between advanced liver fibrosis and mortality is modified by a high-quality diet.

## 1. Introduction

Nonalcoholic fatty liver disease (NAFLD) is the leading cause of chronic liver disease worldwide. The global prevalence of NAFLD is approximately 25.2% and has significantly increased alongside obesity [1]. NAFLD encompasses a wide spectrum of nonalcoholic fatty liver (simple steatosis), nonalcoholic steatohepatitis (NASH), cirrhosis (severe fibrosis), and hepatocellular carcinoma [2]. NAFLD is closely related to metabolic comorbidities, such as overweight or obesity, type 2 diabetes, and metabolic syndrome, which lead to inflammation, altered hepatic glucose and lipid metabolism, and carcinogenesis [3]. Cardiovascular disease (CVD) is the most common cause of death in NAFLD, followed by extrahepatic malignancies, complications related to the liver, and diabetes [4]. Still, it remains uncertain whether NAFLD per se is independently linked to higher risks of all-cause and cause-specific mortality regardless of underlying metabolic dysfunction. Recently, advanced liver fibrosis has received more attention in predicting mortality in NAFLD. Although liver biopsy remains the gold standard for histologic assessment of NAFLD, it cannot be routinely implemented in hospital and population-based studies because of the cost, invasiveness, and expertise required for interpretation [5]. The NAFLD fibrosis score (NFS) and the Fibrosis-4 index (FIB-4) are noninvasive score systems validated to assess advanced fibrosis in people with NAFLD [6]. The cut-off values of NFS > 0.676 and FIB-4 ≥ 2.67 identified the presence of advanced fibrosis in people with NAFLD with a positive predictive value of 82–90% [7] and 80% [8], respectively. In a US population-based study, advanced fibrosis determined by noninvasive panels, such as the NFS and FIB-4, was a significant predictor of all-cause and cardiovascular mortality, whereas NAFLD was not [9]. In a meta-analysis of five cohort studies, increasing the fibrosis stage in NAFLD significantly increased the risks of all-cause and liver-related mortality [10]. A hospital-based cohort study in Korea also showed that NAFLD with liver fibrosis determined by FIB-4 was associated with increased risks of all-cause and cause-specific mortality [11]. However, studies examining the relationship between mortality and advanced liver fibrosis are limited in an Asian population with NAFLD.

Achieving weight loss through lifestyle modification is the cornerstone of managing NAFLD of any severity [5]. Regarding liver fibrosis, weight loss of at least 10% can result in fibrosis regression or stabilization in people with NASH [12]. A calorie-restricted diet with a daily reduction of 500–1000 kcal is generally recommended for significant weight loss to treat NAFLD [5,12]. On the other hand, a healthy eating pattern may also be important in the management of NAFLD. In a randomized-controlled trial (RCT), people with NAFLD who followed the Mediterranean diet experienced improved liver steatosis and insulin sensitivity without weight loss [13]. Accumulating data support that diets with reduced amounts of free sugars, refined carbohydrates, and saturated fat can be helpful in the treatment of NAFLD [14,15]. Data from the Global Burden of Disease 2017 showed that a high intake of sugar-sweetened beverages, red meat, and trans-fatty acids, and a low intake of nuts, seeds, and milk, were significantly associated with liver-related deaths in NAFLD [16]. However, a cohort study of data from the third National Health and Nutrition Examination Survey (NHANES) found no association between high diet quality and a reduced risk of all-cause mortality in people with NAFLD [17]. The effect of diet quality on mortality in those with NAFLD remains inconclusive. Therefore, more evidence is needed to elucidate the role of diet quality in reducing mortality in NAFLD, especially advanced liver fibrosis.

In the present study, we tested the association between advanced hepatic fibrosis and all-cause and cardiovascular mortality in people with NAFLD using a nationally representative Korean cohort. We also tested the hypothesis regarding whether diet quality modified the observed associations. 

## 2. Materials and Methods

### 2.1. Study Population

We analyzed data from the Korea National Health and Nutrition Examination Survey (KNHANES) 2007–2015. The KNHANES is a nationally representative, cross-sectional examination and survey used to assess the health and nutritional status of Korean people. The Korea Disease Control and Prevention Agency (KDCA) has conducted the KNHANES annually since 2007. The detailed methods of the KNHANES are described elsewhere [18]. In brief, the KNHANES includes health interviews, health examinations, laboratory tests and anthropometric measures, and nutrition surveys. The KNHANES data were linked to the 2007–2019 Cause of Death Statistics for individuals aged ≥ 19 years [19]. Of 73,353 individuals enrolled in the KNHANES 2007–2015, 51,575 participants agreed to link their data to the Cause of Death Statistics. Among these participants, we excluded those (1) aged < 20 years (*n* = 567); (2) who were pregnant (*n* = 289); (3) who had missing information on sample weights (*n* = 5949), (4) who were positive for serologic markers of hepatitis B (*n* = 1505) or C (*n* = 154) viruses; (5) who had excessive alcohol consumption, defined as more than seven drinks twice a week for men and more than five for women (*n* = 4124); (6) who had a history of CVD (*n* = 1939) or cancer (*n* = 1396); and (7) who died within the first year of follow-up (*n* = 121). Ultimately, 35,531 adults with suspected NAFLD, excluding competing etiologies of chronic liver disease, were included in this study. The study was approved by the Institutional Review Board of Korea University (KUIRB-2020-0291-01).

### 2.2. Definition of Advanced Liver Fibrosis

NFS and the FIB-4 were used to determine the severity of liver fibrosis. NFS was calculated as −1.675 + 0.037 × age (years) + 0.094 × body mass index (kg/m^2^) + 1.13 × impaired fasting glucose or diabetes (1, if yes; 0, if no) + 0.99 × [aspartate transaminase (AST)/alanine transaminase (ALT) ratio] − 0.013 × platelet (×10^9^/L) − 0.66 × albumin (g/dL) [7]. FIB-4 was calculated as age (years) × AST (U/L)/[platelet count (×10^9^/L) × √ALT (U/L)] [20]. Serum albumin was not included to calculate NFS due to the lack of data in the KNHANES [21]. Advanced liver fibrosis was defined as either the highest quartile value of NFS (≥1.54) [21] or FIB-4 ≥ 2.67 [22]. Although the cut-off value of NFS > 0.676 was used in other studies [7], the new cut-off point (NFS ≥ 1.54 as the highest quartile value) was chosen in this analysis as the previous cut-off value (NFS > 0.676) was not suitable for Korean population as well as the lack of serum albumin used in calculating NFS.

### 2.3. Mortality Assessment

Participants were followed from baseline (survey entry) through the date of death or to the end of follow-up on 31 December 2019, whichever occurred first. Date and causes of death were ascertained by reviewing death certificates and medical records provided by Statistics Korea. The underlying causes of death were classified according to the codes from the International Classification of Diseases, 10th version (ICD-10). We identified all-cause mortality (3426 deaths), cardiovascular mortality (I00-I99; 778 deaths), and cancer mortality (C00-D48; 1101 deaths).

### 2.4. Covariate Assessment

Demographic information, including age, sex, education level, residence area, and household income status, was obtained from standardized health questionnaires. Education level was categorized as elementary school or below, middle school, high school, and university or above. Household income status was divided into 4 groups: lowest, lower-middle, upper-middle, and highest. Smoking and alcohol consumption were reported as current smoking or non-smoking and current drinking or non-drinking, respectively, via self-reporting at the time of the survey. Physical activity was assessed using metabolic equivalents (METs), calculated by multiplying the intensity of physical activity (2.4 for light, 5.0 for moderate, and 7.5 for vigorous) and the weekly frequency. Blood samples were taken in the morning after fasting for at least 8 h. Fasting plasma glucose (FPG), glycosylated hemoglobin (HbA1c), high-density lipoprotein (HDL) cholesterol, low-density lipoprotein (LDL) cholesterol, AST, and ALT were measured. Blood pressure was measured using a sphygmomanometer, and the final systolic blood pressure (SBP) and diastolic blood pressure (DBP) were defined as the average of second and third estimations. Hypertension was defined as SBP ≥ 140 mm Hg, DBP ≥ 90 mm Hg, a previous diagnosis of hypertension, or taking anti-hypertensive drugs. Diabetes mellitus was defined as FPG ≥ 126 mg/dL, HbA1c ≥ 6.5%, a previous diagnosis of diabetes, or taking anti-diabetic drugs. Hypercholesterolemia was defined as total cholesterol ≥ 240 mg/dL or taking lipid-lowering drugs.

### 2.5. Diet Quality Assessment

Nutrition status was estimated by a 24 h dietary recall. We used the Korean Healthy Eating Index (KHEI), which was developed by the KDCA to evaluate comprehensive diet quality. The KHEI consists of 14 components in 3 categories, including adequacy, moderation, and balance. The detailed methods of KHEI are described elsewhere [23,24]. Among the components, we did not include a percentage of energy from saturated fatty acids because of limited data on dietary fat subtypes. The total KHEI was scored from 0 to 90 points. We previously calculated the KHEI scores from the KNHANES data and categorized them into three groups as follows: low (KHEI ≤ 45; lowest quartile (Q1)), moderate (KHEI 46–60; middle quartiles (Q2–Q3)), and high (KHEI ≥ 61; highest quartile (Q4)) [24]. To examine the protective effect of high diet quality, the KHEI scores were classified into two groups: low and moderate diet quality and high diet quality [25,26,27]. In the present study, we used the cut-off values of previous study, so low and moderate diet quality was defined as ≤60 KHEI and high diet quality was defined as ≥61 KHEI. 

### 2.6. Statistical Analysis

The KNHANES is based on the multi-stage clustered probability design. The complex sampling design was considered during data analysis, including the sample weights, cluster, and strata [18]. The characteristics of the participants are presented as the mean ± standard error for continuous variables and the number and percentage for categorical variables. The student’s *t*-test and chi-square test were conducted to compare continuous and categorical variables. The Cox proportional hazards model was used to estimate the hazard ratios (HRs) and 95% CIs for all-cause and cardiovascular mortality according to the presence of advanced liver fibrosis determined by NFS and FIB-4. To investigate the combined effects of NFS and FIB-4 on mortality, we also compared three groups according to the combination of NFS and FIB-4, as low NFS + low FIB-4, low NFS + high FIB-4 or high NFS + low FIB-4, and high NFS + high FIB-4. We adjusted for potential confounders at baseline. Model 1 was adjusted for age (years), sex (male/female), residential area (urban/rural), education level (elementary school or below/middle school/high school/university or above), household income status (lowest/lower-middle/higher-middle/highest), smoking status (non-smoker/current smoker), alcohol consumption (non-drinker/current drinker), physical activity (METs), total energy intake (kcal), and waist circumference (cm). Model 2 were adjusted further for the prevalence of hypertension, diabetes mellitus, and hypercholesterolemia. We also conduced stratified analyses on diet quality to examine the associations between advanced liver fibrosis and mortality. All statistical analyses were performed using Stata SE 13.0 (Stata Corp, College Station, TX, USA), and *p*-values of <0.05 were considered statistically significant.

## 3. Results

### 3.1. Characteristics of the Study Population

**Figure 1:**
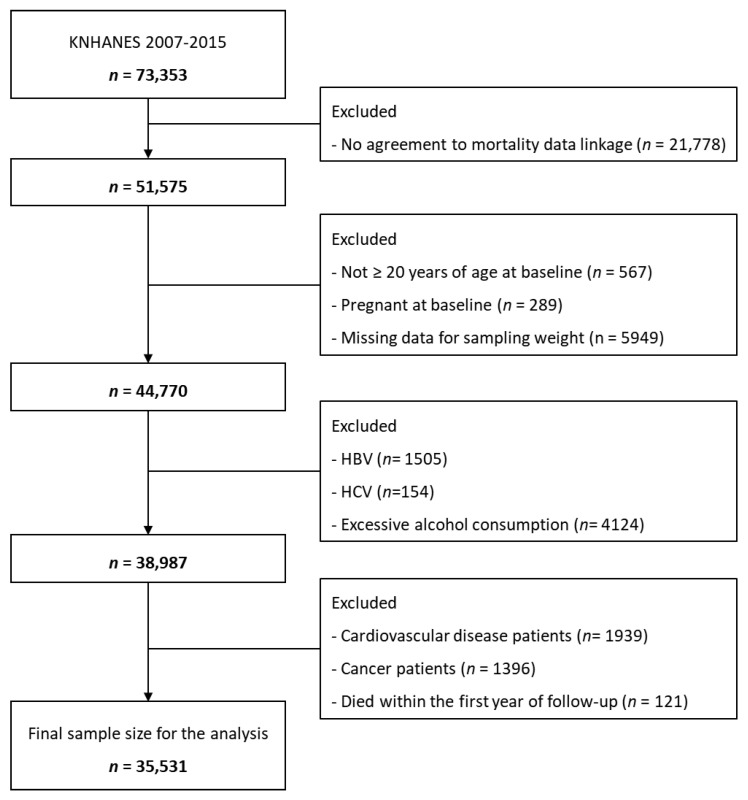
Flow diagram of study population.

A total of 35,531 participants (12,839 men and 22,692 women) were included in this study (Figure 1). The mean age was 45.2 years. Table 1 shows the baseline characteristics of the study population with or without advanced liver fibrosis. The prevalence of advanced liver fibrosis was 23.7% and 2.1% as assessed by NFS and FIB-4, respectively. The participants with advanced liver fibrosis were more likely to be older and men, to live in a rural area, and to have a low education level and low income status. Current drinking and physical activity levels were lower in those with advanced liver fibrosis. They had higher values of cardiometabolic parameters such as waist circumferences, SBP, DBP, FPG, HbA1c, total cholesterol, triglycerides, LDL cholesterol, and aminotransferases. The proportions of hypertension, diabetes mellitus, and hypercholesterolemia were higher in the participants with advanced liver fibrosis than in those without advanced liver fibrosis.

### 3.2. Advanced Liver Fibrosis and Mortality

Table 2 shows the association of advanced liver fibrosis determined by NFS and FIB-4 with mortality. A total of 3426 deaths were reported during a mean 8.1 years of follow-up. Compared with the low NFS and low FIB-4 groups, the risk of all-cause mortality significantly increased in the high NFS and high FIB-4 groups even after adjusting for confounders. In model 2, the HRs for all-cause mortality were 1.31 (95% CI 1.11–1.54) and 1.72 (95% CI 1.35–2.19), respectively. An increased risk of cardiovascular mortality was observed in the high NFS + high FIB-4 groups compared with the low NFS + low FIB-4 groups (Table 3). After adjusting confounders, it became insignificant in the high NFS group (in model 2: HR 1.29, 95% CI 0.93–1.78), whereas the significance remained in the high FIB-4 group (in model 2: HR 1.82, 95% CI 1.17–2.84). The HRs for all-cause and cardiovascular mortality were estimated to investigate the combined effects of NFS and FIB-4 on mortality. Compared with the low NFS + low FIB-4 group, the high NFS + high FIB-4 group significantly increased the risks of all-cause and cardiovascular mortality. In model 2, the adjusted HRs were 1.85 (95% CI 1.42–2.43) and 2.04 (95% CI 1.23–3.39), respectively. There were no differences in all-cause and cardiovascular mortality between either the low NFS + high FIB-4 or high NFS + low FIB-4 group and the low NFS + low FIB-4 group.

### 3.3. Stratified Analysis by Diet Quality

Table 4 presents the association between advanced liver fibrosis and mortality according to diet quality. In the low and moderate diet quality group (KHEI ≤ 60), the participants with advanced liver fibrosis were associated with significantly higher risks of all-cause and cardiovascular mortality than those without advanced liver fibrosis. The HRs for all-cause mortality in model 2 were 1.27 (95% CI 1.06–1.53) and 1.79 (95% CI 1.38–2.35) when assessed by NFS and FIB-4, respectively. The corresponding HRs for cardiovascular mortality were 1.46 (95% CI 1.03–2.09) and 2.00 (95% CI 1.25–3.20). In contrast, no significant associations between advanced liver fibrosis and all-cause or cardiovascular mortality were observed in the high diet quality group (KHEI ≥ 61) in model 1 and model 2. However, an increase in the KHEI scores was associated with lower risks of all-cause and cardiovascular mortality in the overall study population (Table S1).

## 4. Discussion

In this large-scale population-based study, advanced liver fibrosis determined by NFS and FIB-4 was significantly associated with increased risks of all-cause and cardiovascular mortality after adjusting for potential confounders. These results were also similar when NFS and FIB-4 were combined to identify advanced liver fibrosis. Since we excluded competing etiologies of coexisting chronic liver disease, most study participants with liver fibrosis were considered to have NAFLD. Intriguingly, the association between advanced liver fibrosis and mortality was observed in the low and moderate diet quality group but not in the high diet quality group. Our findings suggest that advanced liver fibrosis is an independent factor for all-cause and cardiovascular mortality risk in people with NAFLD, and lifestyle modification with a high-quality diet may help reduce mortality.

In our study, we defined advanced liver fibrosis as the highest quartile of NFS (≥1.54) or FIB-4 ≥ 2.67. The higher cut-off value of NFS in our study might be partly explained by the lack of serum albumin in calculating NFS [21]. We also combined NFS and FIB-4 to better distinguish the participants with advanced liver fibrosis from those without advanced liver fibrosis. A cross-sectional study discovered that the combination of NFS and FIB-4 improved the sensitivity and specificity in identifying liver fibrosis, as detected by magnetic resonance elastography, among people with NAFLD [28]. Previous studies have shown that liver fibrosis assessed using NFS and FIB-4 is an important predictor of overall mortality in people with ultrasonography (USG)-diagnosed [9,29] or biopsy-proven NAFLD [30,31,32]. In people with heart failure with preserved ejection fraction, liver fibrosis with NFS ≥ 1.56 upon inclusion of serum albumin was associated with an increased risk of all-cause mortality [33]. Thus, we reliably assessed advanced liver fibrosis using NFS and FIB-4 to investigate its association with mortality.

In our study, advanced liver fibrosis was independently associated with increased risks of all-cause and cardiovascular mortality in people with suspected NAFLD. This finding was consistent after adjusting for cardiometabolic parameters and metabolic co-morbidities. In a US study using NHANES data, advanced fibrosis determined by noninvasive markers was a significant predictor of all-cause mortality, mainly from CVD, in adults with NAFLD detected by USG during a median follow-up of 14 years [9]. Several pathophysiological mechanisms by which NAFLD and liver fibrosis promote the development of CVD have been proposed [34,35,36]. However, it remains uncertain whether they are mediators or bystanders of CVD. Current evidence indicates that NAFLD or liver fibrosis by itself can contribute to and accelerate the development of CVD [36]. In a meta-analysis of 36 observational studies, NAFLD was associated with an increased risk of fatal or nonfatal CVD, and the risk further increased with advanced liver disease, particularly at higher stage of fibrosis, independent of cardiometabolic risk factors [37]. A recent study of the NHANES showed that advanced fibrosis in metabolic-dysfunction-associated fatty liver disease was associated with a higher risk of all-cause mortality than NAFLD after adjusting for known risk factors, which suggests that an unfavorable metabolic milieu increases mortality [38]. In our study, the participants with advanced liver fibrosis were older and had higher probabilities of traditional risk factors and social determinants of health influencing incident CVD than those without advanced liver fibrosis. On the other hand, a nationwide cohort study in Sweden with biopsy-confirmed NAFLD reported that worsening NAFLD histopathology increased overall mortality, and excess mortality was primarily from extrahepatic malignancies and cirrhosis rather than CVD [39]. In a meta-analysis of 13 cohort studies, biopsy-confirmed liver fibrosis was a key prognostic factor for all-cause and liver-related mortality in people with NAFLD with or without NASH [40]. Various factors, such as metabolic abnormalities and the stage and clinical course of fibrosis, affect the all-cause and cause-specific mortality of people with NAFLD. Therefore, a better understanding of the pathophysiology and phenotypes of NAFLD is needed to clarify the relationship between advanced liver fibrosis and mortality.

Interestingly, we found that the association of advanced liver fibrosis with all-cause and cardiovascular mortality was prominent in the participants with low and moderate diet quality but disappeared in those with high diet quality. Our findings are in line with the results of previous observational studies that reported an inverse association between dietary composition modification and fibrosis progression in people with NAFLD [41,42]. It has been shown that unhealthy dietary habits could be associated with fibrosis progression in NASH [41,42]. We evaluated diet quality using the KHEI in the present study. A recent cross-sectional study in Korea demonstrated inequalities in diet quality assessed by the KHEI among age groups and socioeconomic status despite modest improvement in diet quality in the past decade, which might be related to unfavorable cardiometabolic risk factor profiles [24]. Higher diet quality does not hinge on any single component of the diet but rather indicates adherence to healthy eating patterns that emphasize a variety of healthier foods while limiting the intake of sugars and fats from ultra-processed foods and beverages [43,44,45]. Several studies suggest that various food items and nutrients have been linked with an increased risk of NAFLD and the severity of NASH [46]. For example, diets rich in saturated fat increase both liver fat and insulin resistance, and replacing saturated fats with monounsaturated fatty acids may improve NAFLD [47,48]. The Mediterranean diet may reduce liver fat even without weight loss and reduced carbohydrate intake [13]. However, the effect of a specific dietary intervention has not yet been established in people with NAFLD and advanced liver fibrosis. In an RCT, 800 IU/day of vitamin E improved liver histopathology in nondiabetic adults with biopsy-proven NASH with no improvement in fibrosis [49]. In this respect, the modifying effect of diet quality in our study may underscore the importance of regular consumption of a balanced diet and suggests its role as a modifiable factor to reduce the mortality risk in advanced liver fibrosis.

Recently, gut dysbiosis has been revealed as the pathogenic factor of NAFLD. Gut dysbiosis increases gut permeability and translocation of lipopolysaccharide (LPS) from the gastrointestinal tract into systemic circulation [50,51]. In people and mice with NASH, LPS could promote low-grade endotoxemia, localize in the hepatocytes, and elicit liver damage [52]. Dietary intervention may improve gut barrier function and decrease circulating LPS levels, thereby delaying the progression of the natural history of NAFLD [50,53]. Gut dysbiosis also induces the production of reactive oxygen species, amplifies hepatic oxidative stress, and involves immune cell-mediated inflammation in NAFLD [36,54]. Mounting evidence shows that dietary intervention strongly influences systemic inflammation and oxidative stress via gut microbiota-derived metabolites such as trimethylamine N-oxide [55]. RCTs are needed to investigate whether a high-quality diet with beneficial effects on gut dysbiosis reduces all-cause or cause-specific mortality in people with NAFLD and liver fibrosis.

The strength of our study is that, to the best of our knowledge, this is the first study to evaluate an independent association of noninvasive biomarkers of liver fibrosis with all-cause and cardiovascular mortality in a Korean population with a sufficient follow-up duration. In addition, we investigated the effect of diet quality on the association between advanced liver fibrosis and mortality using a validated index. However, our study has several limitations. First, we did not identify people with NAFLD using imaging tests or biopsies. Although we excluded etiologies of common chronic liver disease, individuals with non-NAFLD could be included in the analysis. Second, we used only two noninvasive scoring systems (NFS and FIB-4). There was discordance in cardiovascular mortality between the high NFS and high FIB-4 groups. However, several studies have reported that FIB-4 is superior to other noninvasive markers to identify liver fibrosis [8] and the risk of cardiovascular mortality in people with NAFLD [29]. Third, since all potential confounding factors, such as the fibrosis stage and progression or regression of NAFLD histopathology, were not adjusted for, residual confounding might influence the results of our study. Fourth, nutrition status evaluation in our study was restricted to a single 24 h dietary recall, which might be too short to evaluate diet quality and reflect individuals’ food preference. There could also be some bias because the 24-h dietary recall relied on respondents’ memory [56]. Nevertheless, the 24-h dietary recall which is administered by skilled technicians using standardized protocols has been validated and provides quantitative estimates of comprehensive food consumption in KNHANES, a large-scale nutrition survey. Finally, information on the treatment for metabolic comorbidities was unavailable.

## 5. Conclusions

In conclusion, our population-based cohort study showed that advanced hepatic fibrosis determined by NFS and FIB-4 was an independent risk factor for all-cause and cardiovascular mortality in Korean adults with NAFLD. In addition, the association between advanced liver fibrosis and mortality was modified by a high-quality diet. Therefore, lifestyle modification focused on diet quality may be a promising way to reduce mortality in this population.

## Figures and Tables

**Table 1:** Baseline characteristics of the study participants with or without advanced liver fibrosis.

	All	NFS			FIB-4		
		<1.54	≥1.54	*p*-Value	<2.67	≥2.67	*p*-Value
*N* (%)	35,531	22,536 (76.3)	7009 (23.7)		30,330 (97.9)	638 (2.1)	
weighted *N* (weighted %)	29,419,312	20,202,666 (82.6)	4,253,682 (17.4)		25,488,644 (98.7)	344,085 (1.3)	
Age, years	45.2 ± 0.1	41.2 ± 0.1	61.7 ± 0.2	<0.001	44.5 ± 0.1	67.1 ± 0.7	<0.001
Male, *N* (%)	12,839 (45.3)	7561 (44.8)	3179 (49.9)	<0.001	11,021 (46.0)	341 (54.9)	<0.001
Residential area, *N* (%)							
Urban Rural	23,472 (71.0) 12,059 (29.0)	15,408 (72.6) 7128 (27.4)	4343 (66.7) 2666 (33.3)	<0.001	20,339 (71.6) 9991 (28.4)	378 (63.6) 260 (36.4)	<0.001
Education level, *N* (%)							
≤Elementary school graduate Middle school graduate High school graduate ≥University graduate	9114 (18.0) 3601 (9.3) 11,097 (38.2) 9993 (34.5)	4023 (11.5) 2057 (7.9) 8192 (40.9) 8057 (39.7)	3316 (42.3) 1047 (15.6) 1529 (25.5) 914 (16.6)	<0.001	7187 (16.4) 3102 (9.2) 9829 (38.6) 9072 (35.9)	352 (54.6) 77 (13.6) 118 (23.1) 52 (8.7)	<0.001
Household income level, *N* (%)							
Lowest Lower-middle Upper-middle Highest	7176 (15.4) 8868 (25.7) 9441 (29.4) 9525 (29.5)	3129 (11.2) 5538 (25.4) 6715 (31.4) 6960 (31.9)	2409 (28.8) 1833 (26.0) 1396 (23.6) 1277 (21.5)	<0.001	5571 (14.1) 7566 (25.6) 8359 (30.1) 8502 (30.3)	293 (42.6) 179 (29.2) 87 (15.9) 67 (12.3)	<0.001
Current smoker, *N* (%)	5353 (20.5)	3625 (21.2)	911 (15.6)	<0.001	4679 (20.8)	98 (20.2)	0.785
Current drinker, *N* (%)	16,055 (53.0)	11,092 (55.3)	2913 (46.5)	<0.001	14,139 (53.8)	272 (46.8)	<0.001
METs, min/wk	2015.5 ± 28.6	2004.8 ± 31.6	1884.6 ± 52.9	0.021	2011.8 ± 29.0	1733.6 ± 155.3	0.037
KHEI	52.7 ± 0.1	52.7 ± 0.1	54.2 ± 0.2	<0.001	52.9 ± 0.1	51.2 ± 0.6	0.004
Cardiometabolic parameters							
Body mass index, kg/m^2^	23.58 ± 0.03	23.29 ± 0.03	24.95 ± 0.06	<0.001	23.59 ± 0.03	23.40 ± 0.19	0.353
Waist circumference, cm	80.6 ± 0.1	79.3 ± 0.1	85.7 ± 0.2	<0.001	80.5 ± 0.1	83.2 ± 0.5	<0.001
Systolic BP, mmHg	116.4 ± 0.1	114.1 ± 0.1	126.0 ± 0.3	<0.001	116.1 ± 0.1	127.5 ± 0.9	<0.001
Diastolic BP, mmHg	75.3 ± 0.1	75.1 ± 0.1	77.2 ± 0.2	<0.001	75.5 ± 0.1	74.4 ± 0.5	0.038
Fasting glucose, mg/dL	96.8 ± 0.2	93.7 ± 0.1	110.9 ± 0.4	<0.001	96.8 ± 0.2	106.2 ± 1.7	<0.001
HbA1c, %	5.78 ± 0.01	5.65 ± 0.01	6.26 ± 0.02	<0.001	5.77 ± 0.01	6.08 ± 0.07	<0.001
Total cholesterol, mg/dL	187.8 ± 0.3	187.3 ± 0.3	191.3 ± 0.5	<0.001	188.0 ± 0.3	177.9 ± 1.6	<0.001
Triglycerides, mg/dL	127.2 ± 0.7	122.1 ± 0.8	147.8 ± 1.8	<0.001	127.2 ± 0.8	137.8 ± 5.1	0.031
HDL cholesterol, mg/dL	49.6 ± 0.1	50.2 ± 0.1	47.2 ± 0.2	<0.001	49.7 ± 0.1	48.7 ± 0.7	0.097
LDL cholesterol, mg/dL	113.6 ± 0.2	113.4 ± 0.3	115.7 ± 0.5	<0.001	113.7 ± 0.2	103.6 ± 1.7	<0.001
AST, IU/L	21.5 ± 0.1	20.5 ± 0.1	24.6 ± 0.2	<0.001	21.0 ± 0.1	45.2 ± 2.0	<0.001
ALT, IU/L	21.5 ± 0.1	21.1 ± 0.2	21.8 ± 0.3	0.014	21.2 ± 0.1	30.3 ± 1.6	<0.001
Metabolic comorbidities							
Hypertension	9505 (22.2)	4444 (16.2)	3562 (48.9)	<0.001	7878 (21.4)	327 (52.4)	<0.001
Diabetes mellitus	3344 (8.7)	1154 (4.6)	1815 (26.7)	<0.001	3006 (8.6)	130 (23.5)	<0.001
Hypercholesterolemia	4420 (12.0)	2770 (10.6)	1392 (20.0)	<0.001	4087 (12.1)	82 (14.8)	0.125

The analyses took into consideration for the complex sampling design (sample weight, cluster, and strata), and the weighted mean values and percentages are presented. Values are presented as the mean ± standard error for continuous variables and as the number and percentage for categorical variables. *p*-values are determined using the student’s *t*-test for continuous variables and the chi-squared test for categorical variables assessing the difference according to the presence of advanced liver fibrosis. ALT, alanine transaminase; AST, aspartate transaminase; BP, blood pressure; FIB-4, fibrosis-4 index; HDL, high-density lipoprotein; KHEI, Korean healthy eating index; LDL, low-density lipoprotein; METs, metabolic equivalent of tasks; *N*, Number; NFS, NAFLD fibrosis score.

**Table 2:** Association between advanced liver fibrosis and mortality according to NFS and FIB-4.

	Weighted Total (*N*)	Weighted Event (*N*)	Weighted Follow-Up (PY)	Weighted Incidence Rate (per 1000 PY)	Model 0: HR (95% CI)	Model 1: HR (95% CI)	Model 2: HR (95% CI)
NFS							
All-cause mortality							
<1.54	20,202,666	245,688	159,100,000	1.54	1.00 (ref)	1.00 (ref)	1.00 (ref)
≥1.54	4,253,682	356,136	31,190,946	11.42	7.71 (6.63–8.97)	1.31 (1.11–1.54)	1.20 (1.01–1.43)
Cardiovascular mortality							
<1.54	20,202,666	52,108	159,100,000	0.33	1.00 (ref)	1.00 (ref)	1.00 (ref)
≥1.54	4,253,682	82,524	31,190,946	2.65	8.44 (6.25–11.39)	1.31 (0.94–1.83)	1.29 (0.93–1.78)
FIB-4							
All-cause mortality							
<2.67	25,488,644	587,982	197,700,000	2.97	1.00 (ref)	1.00 (ref)	1.00 (ref)
≥2.67	344,085	72,898	2,325,029	31.35	11.24 (9.10–13.89)	1.72 (1.35–2.19)	1.72 (1.35–2.18)
Cardiovascular mortality							
<2.67	25,488,644	133,112	197,700,000	0.67	1.00 (ref)	1.00 (ref)	1.00 (ref)
≥2.67	344,085	18,468	2,325,029	7.94	12.60 (8.43–18.83)	1.88 (1.23–2.88)	1.82 (1.17–2.84)

The analyses took into consideration the complex sampling design (sample weight, cluster, and strata), and the weighted values are presented. The hazard ratios (HRs) and 95% CIs were calculated using the Cox proportional hazards model. FIB-4, fibrosis-4 index; NFS, NAFLD (nonalcoholic fatty liver disease) fibrosis score; PY, person-years; ref, reference. Model 0: Crude model. Model 1: Adjusted for age, sex, residential area, education level, household income status, smoking status, alcohol consumption, metabolic equivalent of task, total energy intake, and waist circumference. Model 2: As Model 1 with further adjustments for the prevalence of hypertension, diabetes mellitus, and hypercholesterolemia.

**Table 3:** Risk of all-cause and cardiovascular mortality according to the combination of NFS and FIB-4.

	Weighted Total (*N*)	Weighted Event (*N*)	Weighted Follow-Up (PY)	Weighted Incidence Rate (per 1000 PY)	Model 0: HR (95% CI)	Model 1: HR (95% CI)	Model 2: HR (95% CI)
Low NFS + Low FIB-4	20,195,527	244,931	159,100,000	1.54	1.00 (ref)	1.00 (ref)	1.00 (ref)
Low NFS + High FIB-4 or High NFS + Low FIB-4	3,944,350	292,935	29,080,373	10.07	6.81 (5.82–7.96)	1.23 (1.04–1.46)	1.12 (0.94–1.33)
High NFS + High FIB-4	312,957	63,958	2,143,873	29.83	20.88 (16.33–26.69)	1.91 (1.46–2.51)	1.85 (1.42–2.43)
Low NFS + Low FIB-4	20,195,527	52,108	159,100,000	0.33	1.00 (ref)	1.00 (ref)	1.00 (ref)
Low NFS + High FIB-4 or High NFS + Low FIB-4	3,944,350	66,555	29,080,373	2.29	7.28 (5.36–9.89)	1.22 (0.87–1.72)	1.19 (0.85–1.66)
High NFS + High FIB-4	312,957	15,969	2,143,873	7.45	24.48 (15.04–39.85)	2.04 (1.22–3.15)	2.04 (1.23–3.39)

The analyses took into consideration the complex sampling design (sample weight, cluster, and strata), and the weighted values are presented. The hazard ratios (HRs) and 95% CIs were calculated using the Cox proportional hazards model. Low NFS indicates NFS < 1.54, and high NFS ≥ 1.54. Low FIB-4 indicates FIB-4 < 2.67, and high FIB-4 ≥ 2.67. FIB-4, fibrosis-4 index; NFS, NAFLD (nonalcoholic fatty liver disease) fibrosis score; PY, person-years; ref, reference. Model 0: Crude. Model 1: Adjusted for age, sex, residential area, education level, household income status, smoking status, alcohol consumption, metabolic equivalent of task, total energy intake, and waist circumference. Model 2: As Model 1 with further adjustments for the prevalence of hypertension, diabetes mellitus, and hypercholesterolemia.

**Table 4:** Association between advanced liver fibrosis and mortality according to diet quality.

	Low and Moderate Diet Quality (KHEI ≤ 60) (*n* = 26,078)					High Diet Quality (KHEI > 60) (*n* = 10,798)				
	Weighted Event (*N*)	Weighted Follow-Up (PY)	Model 0: HR (95% CI)	Model 1: HR (95% CI)	Model 2: HR (95% CI)	Weighted Event (*N*)	Weighted Follow-Up (PY)	Model 0: HR (95% CI)	Model 1: HR (95% CI)	Model 2: HR (95% CI)
All-cause mortality										
<1.54	200,775	116,900,000	1.00 (ref)	1.00 (ref)	1.00 (ref)	42,827	42,058,152	1.00 (ref)	1.00 (ref)	1.00 (ref)
≥1.54	294,751	21,919,704	8.09 (6.84–9.57)	1.38 (1.15–1.65)	1.27 (1.06–1.53)	60,479	9,221,865	6.83 (4.89–9.53)	1.05 (0.70–1.56)	0.96 (0.62–1.48)
Cardiovascular mortality										
<1.54	41,405	116,900,000	1.00 (ref)	1.00 (ref)	1.00 (ref)	9585	42,058,152	1.00 (ref)	1.00 (ref)	1.00 (ref)
≥1.54	72,379	21,919,704	9.66 (6.90–13.52)	1.51 (1.05–2.17)	1.46 (1.03–2.09)	10,145	9,221,865	5.06 (2.52–10.19)	0.68 (0.31–1.53)	0.71 (0.32–1.56)
All-cause mortality										
<2.67	481,817	144,500,000	1.00 (ref)	1.00 (ref)	1.00 (ref)	104,080	53,041,238	1.00 (ref)	1.00 (ref)	1.00 (ref)
≥2.67	62,175	1,774,654	11.21 (8.84–14.20)	1.74 (1.34–2.26)	1.79 (1.38–2.35)	9817	525,633	10.25 (6.19–16.98)	1.68 (0.98–2.89)	1.33 (0.78–2.25)
Cardiovascular mortality										
<2.67	113,797	144,500,000	1.00 (ref)	1.00 (ref)	1.00 (ref)	18,197	53,041,238	1.00 (ref)	1.00 (ref)	1.00 (ref)
≥2.67	16,935	1,774,654	12.95 (8.41–19.93)	2.03 (1.29–3.19)	2.00 (1.25–3.20)	1533	525,633	9.03 (2.74–29.77)	0.87 (0.29–2.60)	0.88 (0.30–2.63)

The analyses took into consideration the complex sampling design (sample weight, cluster, and strata), and the weighted values are presented. The hazard ratios (HRs) and 95% CIs were calculated using the Cox proportional hazards model. FIB-4, fibrosis-4 index; *N*, number; NFS, NAFLD (nonalcoholic fatty liver disease) fibrosis score; PY, person-years; ref, reference. Model 0: Crude. Model 1: Adjusted for age, sex, residential area, education level, household income status, smoking status, alcohol consumption, metabolic equivalent of task, total energy intake, and waist circumference. Model 2: As Model 1 with further adjustments for the prevalence of hypertension, diabetes mellitus, and hypercholesterolemia.

## Data Availability

Restrictions apply to the availability of some or all data generated or analyzed during this study to preserve patient confidentiality or because they were used under license. The corresponding author will on request detail the restrictions and any conditions under which access to some data may be provided.

## Supplementary Materials

The following supporting information can be downloaded at: https://www.mdpi.com/article/10.3390/nu15061501/s1, Table S1. Risk of all-cause and cardiovascular mortality according to the KHEI.
