# Supplementary material for: The Association between Advanced Liver Fibrosis and Mortality Is Modified by Dietary Quality among Korean Adults: Results from the Korea National Health and Nutrition Examination Survey with Mortality Data
Source: Nutrients. 2023 Mar 21;15(6):1501. doi: 10.3390/nu15061501 (PMC10053070; doi:10.3390/nu15061501)
Supplement: Supplementary file 1 [file nutrients-15-01501-s001.zip › nutrients-2265381-supplementary.pdf]

Supplementary Table S1. Risk of all-cause and cardiovascular mortality according to the KHEI

	Weighted total (N)	Weighted event (N)	Weighted follow-up (PY)	Weighted incidence rate (per 1,000 PY)	HR (95% CI)
**All-cause mortality**					
KHEI score (continuous)	30,556,809	990,859	243,200,000	4.07	**0.99 (0.98–0.99)**
KHEI score (quintiles)					
Q1	6,019,562	300,129	49,765,182	6.03	1.00 (ref)
Q2	6,068,201	234,935	49,816,015	4.72	0.92 (0.78–1.07)
Q3	6,067,652	176,449	49,234,817	3.58	**0.77 (0.64–0.92)**
Q4	6,242,167	166,540	49,004,146	3.40	**0.77 (0.65–0.93)**
Q5	6,159,224	112,805	45,362,542	2.49	**0.67 (0.54–0.83)**
Low and moderate quality (KHEI ≤60)	21,893,576	814,252	178,500,000	4.56	1.00 (ref)
High quality (KHEI ≥61)	8,663,230	176,607	64,690,357	2.73	**0.83 (0.72–0.97)**
**Cardiovascular mortality**					
KHEI score (continuous)	30,556,809	226,275	243,200,000	0.93	**0.98 (0.97–0.99)**
KHEI score (quintiles)					
Q1	6,019,562	73,539	49,765,182	1.48	1.00 (ref)
Q2	6,068,201	51,822	49,816,015	1.04	0.82 (0.60–1.13)
Q3	6,067,652	45,486	49,234,817	0.92	0.78 (0.56–1.10)
Q4	6,242,167	37,484	49,004,146	0.76	0.71 (0.49–1.02)
Q5	6,159,224	17,944	45,362,542	0.40	**0.42 (0.27–0.68)**
Low and moderate quality (KHEI ≤60)	21,893,576	193,219	178,500,000	1.08	1.00 (ref)
High quality (KHEI ≥61)	8,663,230	33,056	64,690,357	0.51	**0.71 (0.51–0.99)**

The analyses took into consideration the complex sampling design (sample weight, cluster, and strata), and the weighted values are presented. The hazard ratio (HRs) and 95% CIs were calculated using the Cox proportional hazards model adjusting for age, sex, residential area, education level, household income status, smoking status, alcohol consumption, metabolic equivalent of task, total energy intake, weight circumference, and the prevalence of hypertension, diabetes mellitus, and hypercholesterolemia. FIB-4, fibrosis-4 index; NFS, NAFLD (non-alcoholic fatty liver disease) fibrosis score; PY, person-year; ref, reference.
